# Altered Intestinal Morphology and Microbiota Composition in the Autism Spectrum Disorders Associated SHANK3 Mouse Model

**DOI:** 10.3390/ijms20092134

**Published:** 2019-04-30

**Authors:** Ann Katrin Sauer, Juergen Bockmann, Konrad Steinestel, Tobias M. Boeckers, Andreas M. Grabrucker

**Affiliations:** 1 Cellular Neurobiology and Neuro-Nanotechnology lab, Dept. of Biological Sciences, University of Limerick, V94PH61 Limerick, Ireland; Ann.Katrin.Sauer@ul.ie; 2 Institute for Anatomy and Cell Biology, Ulm University, 89081 Ulm, Germany; juergen.bockmann@uni-ulm.de (J.B.); tobias.boeckers@uni-ulm.de (T.M.B.); 3 Gerhard-Domagk-Institute of Pathology, Muenster University Medical Center, 48149 Münster, Germany; Konrad.Steinestel@ukmuenster.de; 4 Health Research Institute (HRI), University of Limerick, V94PH61 Limerick, Ireland; 5 Bernal Institute, University of Limerick, V94PH61 Limerick, Ireland

**Keywords:** microbiome, gut, ProSAP2, Phelan McDermid Syndrome, gut–brain interaction, leaky gut, IL-6, SHANK

## Abstract

Autism spectrum disorders (ASD) are a group of neurodevelopmental disorders characterized by deficits in social interaction and communication, and repetitive behaviors. In addition, co-morbidities such as gastro-intestinal problems have frequently been reported. Mutations and deletion of proteins of the SH3 and multiple ankyrin repeat domains (*SHANK*) gene-family were identified in patients with ASD, and *Shank* knock-out mouse models display autism-like phenotypes. SHANK3 proteins are not only expressed in the central nervous system (CNS). Here, we show expression in gastrointestinal (GI) epithelium and report a significantly different GI morphology in *Shank3* knock-out (KO) mice. Further, we detected a significantly altered microbiota composition measured in feces of *Shank3* KO mice that may contribute to inflammatory responses affecting brain development. In line with this, we found higher *E. coli* lipopolysaccharide levels in liver samples of *Shank3* KO mice, and detected an increase in Interleukin-6 and activated astrocytes in *Shank3* KO mice. We conclude that apart from its well-known role in the CNS, SHANK3 plays a specific role in the GI tract that may contribute to the ASD phenotype by extracerebral mechanisms.

## 1. Introduction

SHANK3 (SH3 and multiple ankyrin repeat domains 3, also known as proline-rich synapse-associated protein 2 (ProSAP2)) is a known scaffolding protein of the postsynaptic density (PSD) of glutamatergic excitatory synapses [1,2,3] that has been associated with autism spectrum disorders (ASD) [4,5,6,7]. Further, the Phelan McDermid Syndrome (PMDS/22q13.3 deletion syndrome) is a rare genetic disorder associated with a heterozygous loss of SHANK3 in the majority of patients. Individuals with PMDS show symptoms of the autism spectrum along with mental retardation and muscular hypotonia, and can suffer from seizures and gastrointestinal (GI) problems [8,9,10]. Besides the CNS, SHANK3 is expressed in different levels in many tissues, such as liver, heart, kidney, skeletal muscle [8], and epithelial cells of the GI tract [11,12].

A growing number of studies indicate a role of abnormal development and function of the gastro-intestinal (GI) system as a factor in ASD, with many patients having symptoms associated with GI disorders [13]. Research indicates a link between the dysfunctions associated with ASD and GI problems such as abnormal trace metal uptake, alterations in the microbiome, and immune dysfunction and inflammatory processes [13,14]. In line with this, we have reported expression of SHANK3 in human enterocytes, where SHANK3 was functionally linked to zinc (Zn) transporter levels mediating Zn absorption [12].

The gut harbors a complex community of microbes—the microbiome—that is able to influence, among others, the development of the central nervous system (CNS) [15]. Recently, a study identified a dysregulation of several genera and species of bacteria in the gut and colon of *Shank3* KO mice [16] and the treatment of *Shank3* KO mice with *L. reuteri* led to the attenuation of some ASD-associated behaviors [16]. However, the underlying factors of the altered microbiota composition are currently not well understood.

Thus, here, we made use of a *Shank3αβ* knock-out mouse line that was reported to display ASD-like behavior with abnormal ultrasonic vocalization, repetitive self-grooming, and reduced interest in novel mice in nonsocial versus novel social pairing in the three-chamber test [17,18]. In these animals we performed a detailed analysis of the GI tract including further analyses determining microbiota composition. Our results confirm expression of SHANK3 in the GI epithelium. Further, *Shank3αβ* knock-out mice display an altered GI morphology and, in line with published data [16] we can confirm changes in gut microbiota composition. Altered GI morphology and microbiota composition lead to exaggerated responses to bacterial metabolites and compounds eliciting an immune response [19]. An increase of inflammatory markers has been reported in individuals with ASD and animal models [20,21]. Especially the cytokine Interleukin-6 (IL-6) has been proposed as a biomarker for autism [22] and was shown to be mechanistically linked to the development of autistic behaviors in mice [23,24,25]. Intriguingly, we detected an increase in IL-6 levels in *Shank3αβ* knock-out mice along with increased activation of astrocytes in the frontal cortex of *Shank3αβ* knock-out mice. Astrocyte activation has previously been linked to ASD [26].

## 2. Results

### 2.1. SHANK3 is Expressed in GI Epithelium of Mice

**Figure 1:**
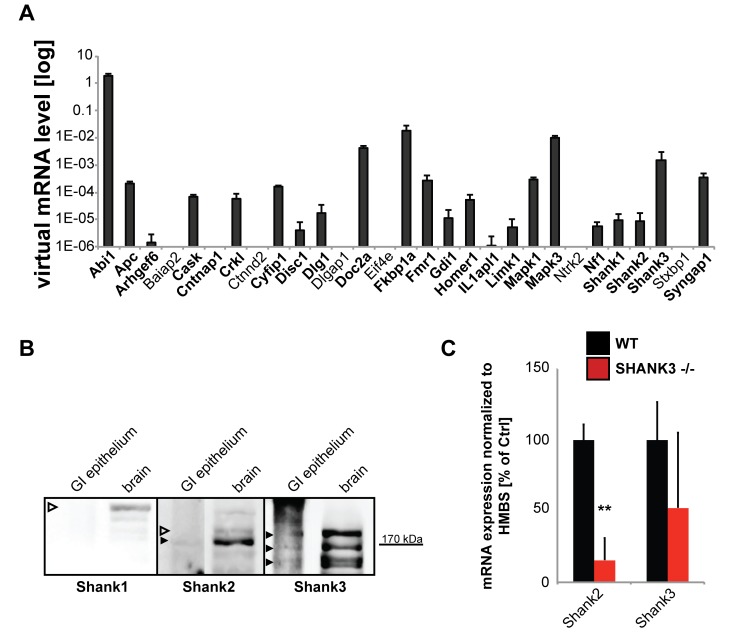
Expression of autism spectrum disorder (ASD)-associated postsynaptic density (PSD) proteins in gut epithelial cells. Several further ASD-associated PSD proteins are expressed in gut epithelial cells. (**A**) Screening of lysate from wild type mice (*n* = 5; used in technical triplicates) from isolated gut epithelium for the expression of “synaptic” ASD-associated genes using qRT-PCR. The genes were selected based on their occurrence at excitatory postsynapses and a reported association with ASD. On mRNA level, expression of all SH3 and multiple ankyrin repeat domains (*Shank)* family members was detected, as well as the expression of several direct interacting proteins such as *Abi1* (Abelson interactor 1), and *Homer1* (Homer protein homolog 1). Furthermore, the expression of *Apc* (Adenomatous-polyposis-coli), *Arhgef6* (Rac/Cdc42 Guanine Nucleotide Exchange Factor (GEF) 6, Alpha-PIX), *Cask* (Calcium/Calmodulin-Dependent Serine Protein Kinase), *Cntnap1* (Contactin Associated Protein 1), *Crkl* (V-Crk Avian Sarcoma Virus CT10 Oncogene Homolog-Like), *Cyfip1* (Cytoplasmic FMR1 Interacting Protein 1), *Disc1* (Disrupted In Schizophrenia 1), *Dlg1* (Discs, Large Homolog 1), *Doc2a* (Double C2-Like Domains, Alpha), *Fkbp1a* (FK506 Binding Protein 1A), *Fmr1* (Fragile X Mental Retardation 1), *Gdi1* (GDP Dissociation Inhibitor 1), *Il1apl1*, *Limk1* (LIM Domain Kinase 1), *Mapk1* and *Mapk3* (Mitogen-Activated Protein Kinase 1 and 3), *Nf1* (Neurofibromin 1), and *Syngap1* (Synaptic Ras GTPase Activating Protein 1) was detected. (**B**) Western Blot analysis for the expression of SHANK family members SHANK1, SHANK2, and SHANK3 using GI epithelium and brain tissue from wild-type mice. Only expression of SHANK2 and SHANK3 was detected on protein level in GI epithelium (full arrows). (**C**) Expression-analysis *Shank2* and *Shank3* in wildtype and *Shank3αβ* KO mice. Significantly lower expression of *Shank2* was found in *Shank3αβ* KO mice (*t*-test, 3 technical replicates from 3 animals per group; *Shank2 p* = 0.0067 (*n* = 3); ** *p* < 0.01).

In the first set of experiments, we investigated the GI system of *Shank3αβ* KO mice that have been characterized in the lab previously [27]. Using the method described by Nik and Carlsson [28], we separated intestinal epithelium from mesenchyme. The purity of the lysate was confirmed by Western Blot analysis of the expression of Vimentin, whose presence would indicate unsuccessful separation of the epithelium, and Cytokeratin 7, which should be found in epithelium but not in mesenchymal cells of the submucosa (Figure S1). Apart from the expression of many ASD-associated genes [29] normally found at synapses in the CNS, we detected mRNA of SHANK family proteins and their “synaptic” interaction partners in GI epithelium (Figure 1A).

On protein level, in wildtype animals, only expression of SHANK2 and SHANK3, but not SHANK1 was found in GI epithelium in mice (Figure 1B). Further, in gut epithelium obtained from *Shank3αβ* KO mice, gene expression of *Shank2* and *Shank3* was decreased in comparison to wild type controls (Figure 1C). Knock-out animals do not show a total loss of *Shank3* due to the expression of the *Shank3γ* isoform that is detected by qRT-PCR primers.

### 2.2. Shank3 KO Mice Show Abnormal GI Morphology

**Figure 2:**
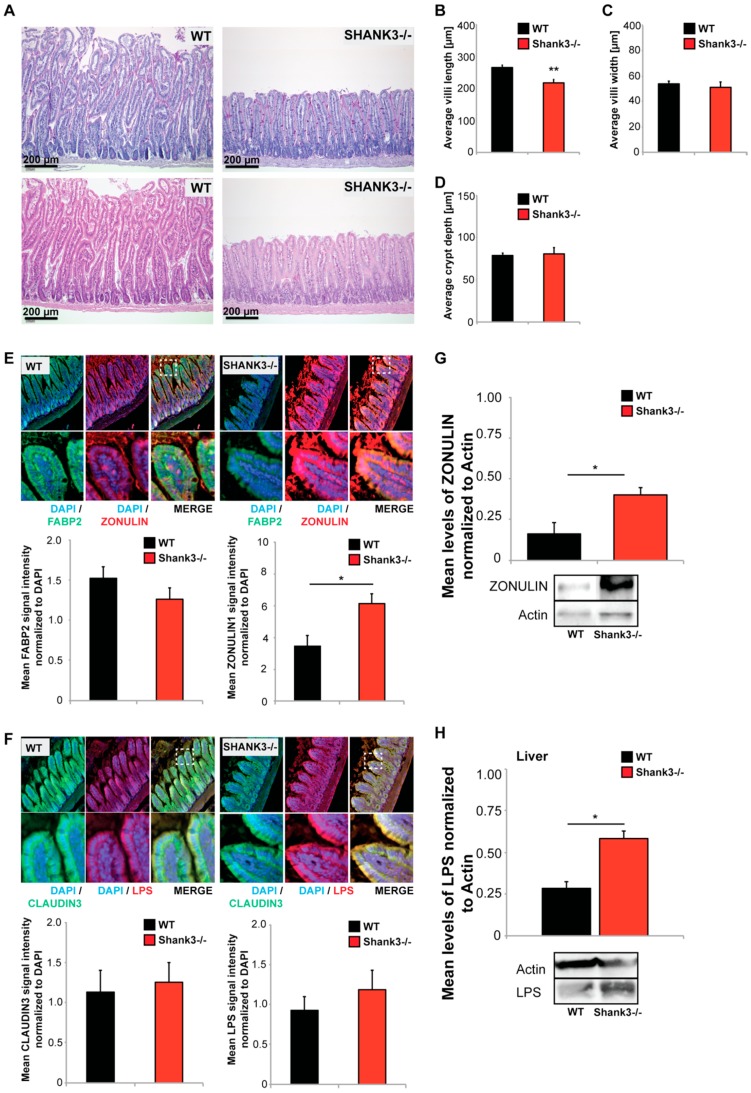
Altered gut morphology in *Shank3αβ* knock-out (KO) mice. (**A**–**D**) Histological evaluation of GI tract from wild type and *Shank3αβ* KO mice. (**A**) Longitudinal cross sections of *Shank3αβ* KO mice and wild type (WT) mice were stained with hematoxylin/eosin (HE) (upper panels) and periodic acid schiff (PAS) reaction (lower panels). Exemplary images are shown. (**B**–**D**) Morphological analysis of (**B**) villi length and (**C**) width, and (**D**) crypt depth reveals a significantly decreased villi length (Mann-Whitney *U*-test, *p* = 0.009; *n* = 5 animals per group) but not width (*p* = 0.534), and normal crypt depth (*p* = 0.983) in *Shank3αβ* KO mice. (**E**,**F**) Immunohistochemistry was performed on 5 mice per group and 5 optic fields of view each from 3 sections per mouse were analyzed. (**E**) A slight but non-significant decrease in FABP2 signal intensity was observed in *Shank3αβ* KO mice compared to wild types (left panel). Significantly higher ZONULIN-1 levels were found in *Shank3αβ* KO mice (right panel) (*t*-test, *p* = 0.0413). (**F**) The levels of CLAUDIN3 and lipopolysaccharide (LPS) were not significantly different between *Shank3αβ* KO mice and wild types in gut epithelium. (**G**) Significantly higher ZONULIN-1 levels in *Shank3αβ* KO mice were confirmed by western blotting using gut epithelium protein lysate (*t*-test, *p* = 0.0434, *n* = 3 per group). (**H**) Protein lysate from liver tissue from WT and *Shank3αβ* KO mice (*n* = 3 per group) were analyzed for *E. coli* LPS levels using Western Blotting. The results show significantly higher LPS levels in the liver of *Shank3αβ β* KO mice (*t*-test, *p* = 0.0452). * *p* < 0.05, ** *p* < 0.01.

*Shank3αβ* KO mice did not show signs of diarrhea, stool blood, weight loss, or increased mortality. However, the analysis of the GI tract of *Shank3αβ* KO mice revealed significantly altered gut morphology (Figure 2A–D). Using paraffin-embedded sections from intestine, we performed histological and morphometric analyses. *Shank3αβ* KO mice show a significantly decreased length, but not width, of small intestinal villi compared to wild type mice (Figure 2B,C). Given that the crypt depth remains unchanged in *Shank3αβ* KO mice (Figure 2D), the ratio between villi length and crypt depth, which is considered normal in a range between 3 and 5, is reduced to below 3 in *Shank3αβ* KO mice.

Further analyses of the GI epithelium using immunohistochemistry and protein biochemistry revealed further alterations. We selected three markers, FABP2 (Intestinal fatty acid-binding protein 2), CLAUDIN3, and ZONULIN1 (Figure 2E,F). FABP2 is a cytosolic protein found in small intestine epithelial cells where it participates in the uptake, intracellular metabolism, and transport of long chain fatty acids. CLAUDIN3 is a cell adhesion protein found at tight junctions between gut epithelial cells. ZONULIN1 is a modulator of tight junctions and alterations in the ZONULIN-regulated pathways have been associated with both intestinal and extra-intestinal inflammatory disorders [30]. Especially a decrease in FABP2 and increase in ZONULIN1 have been proposed as markers of gut dysbiosis and gut permeability integrity [31]. Protein levels were assessed measuring fluorescence intensities. The results reveal slight but not significantly lower levels of FABP2 in *Shank3αβ* KO mice. In contrast, the levels of ZONULIN1 were significantly higher in *Shank3αβ* KO mice compared to wild types (Figure 2E). This result was confirmed using gut epithelial protein lysate and western blotting (Figure 2G). No significant differences were found in CLAUDIN3 levels (Figure 2F).

A loss of intestinal barrier function was reported secondary to upregulation of ZONULIN, which is, to our knowledge, the only known physiological modulator of intercellular tight junctions [32]. Increased intestinal permeability may be responsible for increased translocation of bacterial components and metabolites into the systemic circulation [33]. Given the observed abnormalities in GI epithelium, we therefore investigated next whether the abnormal GI morphology of *Shank3αβ* KO mice facilitates the enrichment of bacterial compounds in the host system.

Lipopolysaccharide (LPS) levels from bacterial origin were not significantly different in the GI epithelium between *Shank3αβ* KO and wild type mice (Figure 2F). Detoxification and degradation of microbial products from gut-derived microbiota is a function of the liver. In the liver, hepatocytes mediate the clearance of endotoxin of intestinal origin [34]. Interestingly, when we analyzed liver samples regarding the levels of bacterial (*E. coli*) LPS, we found a significant increase in liver LPS in *Shank3αβ* KO mice (Figure 2G), hinting at increased LPS absorption (leakiness) of the GI system.

### 2.3. The Microbiome of Shank3 KO Mice Is Altered

**Figure 3:**
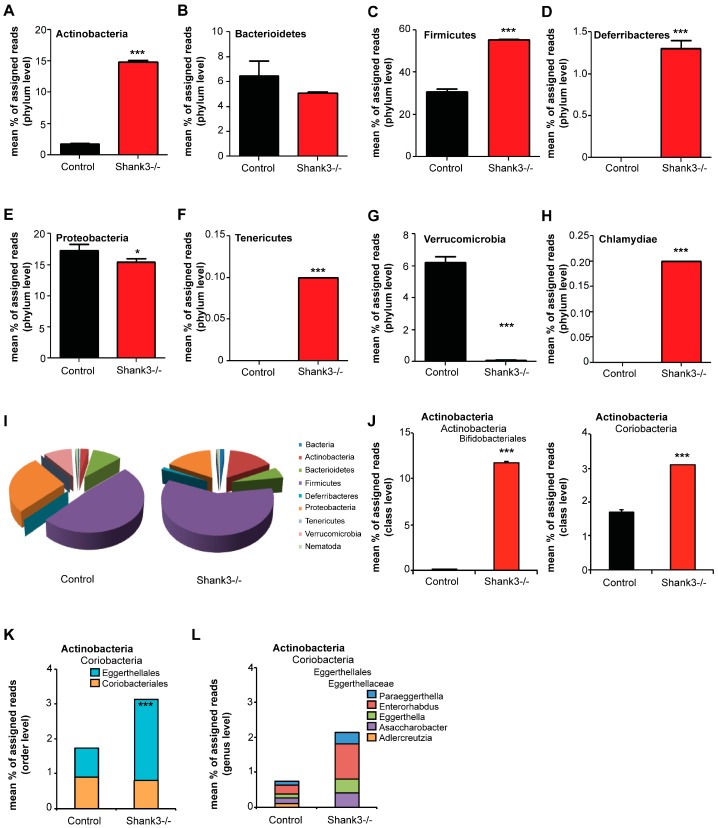
Altered microbiome in *Shank3αβ* KO mice. DNA was extracted from feces from 10 weeks old animals and microbiome analysis was performed using 16S Microbiome Profiling. Feces from four different animals were pooled per sample and three samples per group were analyzed. (**A**) The amount of *Actinobacteria* is significantly higher in feces from *Shank3αβ* KO mice. (**B**) The amount of *Bacterioidetes* is not significantly different between control and *Shank3αβ* KO mice. (**C**) A significant increase in Firmicutes was found in *Shank3αβ* KO mice. (**D**) Bacteria of the phylum *Deferribacteres* were only found in *Shank3αβ* KO mice. (**E**) Significantly reduced levels of Proteobacteria were detected in *Shank3αβ* KO mice. (**F**) Bacteria of the phylum *Tenericutes* were only found in *Shank3αβ* KO mice. (**G**) The amount of *Verrucomicrobia* was significantly lower in *Shank3αβ* KO mice. (**H**) Bacteria of the phylum *Chlamydiae* were only found in *Shank3αβ* KO mice. (**I**) Overview of the identified relative frequencies of different phyla found in control and *Shank3αβ* KO mice. (**J**) The increase in *Actinobacteria* is caused by a significant increase in both classes *Actinobacteria* (order *Bifidobacteriales*) (*t*-test, *p* < 0.0001) and *Coriobacteria* (*t*-test, *p* < 0.0001). (**K**) *Coriobacteria* increase due to a significant higher levels in the order *Eggerthellales*, but not *Coriobacteriales*. (**L**) Within the family *Eggerthellaceae, Adlercreutzia* did not show an increase. The genera *Asaccharobacter, Eggerthella, Enterorhabdus,* and *Paraeggerthella* show significant increase. * *p* < 0.05, *** *p* < 0.001.

Abnormalities in the GI system might translate into persistent changes that may affect several processes and features such as microbiota composition and may cause chronic inflammatory activity. Altered composition of gut microbiota has been reported before in Shank3 KO mice [16]. Thus, in the next set of experiments, we assessed the microbiome of *Shank3αβ* KO mice to confirm the presence of alterations in our mice. Feces from 10 weeks old *Shank3αβ* KO mice were collected and compared to age and gender matched controls. Housing conditions of the mice (bedding material, nesting material, number of animals per cage) were the same between groups and animals were housed side by side in wire cages. DNA was extracted from feces from four mice and pooled to one sample and three samples per group were analyzed using 16s microbiome profiling (Figure 3). The results show significant alterations in the microbiome of *Shankαβ* KO mice compared to Controls (Figure S2A,B). The amount of *Actinobacteria* was significantly higher in feces from *Shank3αβ* KO mice (Figure 3A). While the amount of *Bacterioidetes* (Figure 3B) was not altered, significantly higher levels of *Firmicutes* (Figure 3C) were detected in *Shank3αβ* KO mice. Further, only in *Shank3αβ* KO mice, *Deferribacteres* (Figure 3D), *Tenericutes* (Figure 3F), and *Chlamydiae* (Figure 3H) were found. In contrast, significantly lower levels of *Proteobacteria* (Figure 3E) and *Verrucomicrobia* (Figure 3G) were detected. In general, the phyla *Firmicutes* and *Proteobacteria* dominate the microbiome of control mice, while a shift towards *Firmicutes* and *Actinobacteria* occurs in *Shank3αβ* KO mice (Figure 3I).

Given that an increase in Actinobacteria in the gut of *Shank3* KO mice has been reported before [16], we closer investigated the alterations within this phylum. The increase in *Actinobacteria* was caused by a significant increase in the order *Bifidobacteriales* (class *Actinobacteria*) and *Eggerthellales* (class *Coriobacteria*) (Figure 3J,K). Both orders consist of one detected family, *Bifidobacteriaceae* and *Eggerthellaceae*, respectively. Within the family *Bifidobacteriaceae*, only bacteria of the genus Bifidobacterium were detected. Within the family *Eggerthellaceae*, the genus *Adlercreutzia* did not show an increase, while bacteria of the genus *Asaccharobacter, Eggerthella, Enterorhabdus*, and *Paraeggerthella* increased in abundance (Figure 3L). In particular, the bacteria species *Bifidobacterium pseudolongum*, *Assacharobacter WCA-131-CoC-2*, *Eggerthella YY7918*, and *Enterorhabdus caecimuris* were drivers of this increase (Figure S2C), which have been associated with inflammation and infection of the gastrointestinal tract [35,36]. However, classification on species level using 16S RNA sequencing cannot be done with a high level of confidence and needs to be confirmed by more detailed studies in the future. Another limitation of the performed analysis is that mice have been pooled into three samples, which obscures inter-individual differences. For analysis, we assumed a normal distribution of data. While pooled samples within one group showed great homogeneity, high levels of variability on individual level are not uncommon for microbiota composition.

### 2.4. Altered GI Morphology and Microbiome of Shank3 KO Mice May Be Linked to Increased Inflammatory Marker Expression

One hypothesis that has been proposed for ASD is that GI pathologies such as a “leaky gut” will expose the host to epitopes from microbiota that reside within the gut in altered composition, and thereby produce an immune activation leading to inflammatory responses, which may contribute to CNS pathologies during certain time-windows in development. Altered inflammatory cytokine levels have been reported recently in *Shank3* KO mice [16].

**Figure 4:**
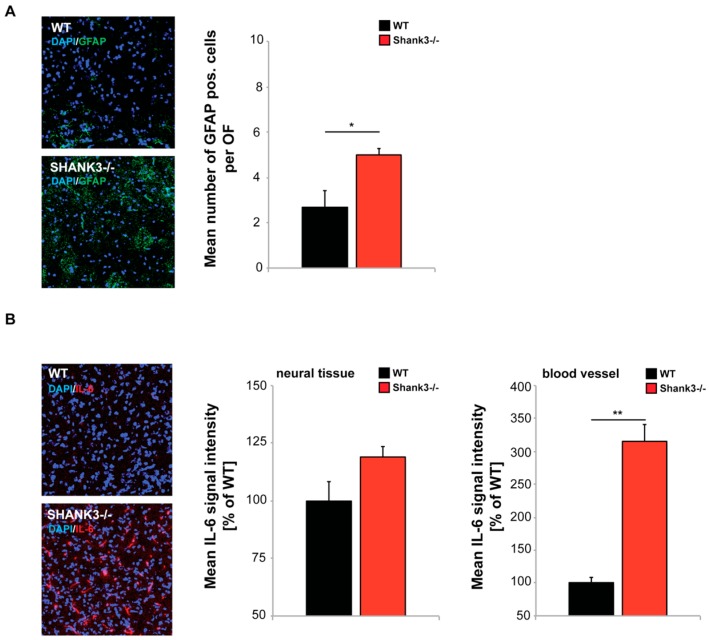
Confocal microscopy images with same acquisition time taken from frontal cortex of brain sections from WT and *Shank3αβ* KO mice (*n* = 3 animals per group) were used to assess the number of activated astrocytes labeled by Glial fibrillary acidic protein (GFAP), and IL-6 levels in neural tissue and blood vessels. DAPI staining was used to visualize cell nuclei. (**A**) Optic fields (OF) of view were analyzed and the number of GFAP positive cells per OF measured. The results show a significantly higher number of activated astrocytes in *Shank3αβ* KO mice (*t*-test, *p* = 0.0407). (**B**) The immunofluorescence of IL-6 was slightly higher in *Shank3αβ* KO mice compared to WT (*t*-test, *p* = 0.1164) in neural tissue and significantly higher in blood vessels (*t*-test, *p* = 0.0014). * *p* < 0.05, ** *p* < 0.01.

Therefore, next, to investigate whether higher intestinal barrier dysfunction of *Shank3αβ* KO mice might translate into increased expression of inflammatory markers, we analyzed the expression of Glial fibrillary acidic protein (GFAP), a marker for astrogliosis, in the cortex of WT and *Shank3αβ* KO mice using immunohistochemistry. Our results reveal a significantly increased number of GFAP positive cells in *Shank3αβ* KO mice (Figure 4A). Further, because of its importance in relation to ASD, we analyzed IL-6 levels in brain sections of *Shank3αβ* KO mice. IL-6 signals resulted from diffuse staining of neural tissue and signals from blood vessels. The immunofluorescence of IL-6 was slightly, but not significantly, higher in neural tissue of *Shank3αβ* KO mice compared to WT (Figure 4B). In contrast, signals in blood vessels were significantly increased in *Shank3αβ* KO mice (Figure 4B) hinting at a systemic increase of IL-6 as previously reported in individuals with ASD.

## 3. Discussion

A growing amount of research reveals abnormalities in the GI system of ASD patients with many of them having symptoms associated with GI disorders. It is likely that these extracerebral alterations contribute to and modify the pathology of ASD. Here, we investigated *Shank3αβ* KO mice and found significant GI abnormalities that translated into altered microbiota composition, increased accumulation of bacterial LPS in liver, and signs of increased immune activation in the periphery and the brain.

The structural and functional integrity of the gastrointestinal mucosal barrier is important for protection from various luminal agents such as acids, enzymes, bacteria, viruses, and toxins. The abnormal GI morphology may lead to downstream effects resulting in the often-reported GI-related symptoms and co-morbidities in ASD [13]. One frequently reported GI alteration in ASD is an altered microbiome. In mammals, intestinal microbiota have a marked influence on health status via gut–brain–microbiota interactions [37,38]. In humans, stool was shown to contain a high bacterial composition, with >90% of sequence data belonging to bacteria [39], with predominately bacteria belonging to two phyla, *Firmicutes* and *Bacteriodetes* [39]. Interestingly, feces of mice also showed mostly bacteria from the phyla *Firmicutes* and *Bacteriodetes* in our analyses. The dysbiosis we observed in *Shank3αβ* KO mice is marked by an increase in microbiota of the phylum *Actinobacteria* and *Firmicutes*, and bacteria from the phylum *Tenericutes*, *Deferribacteres*, and *Chlamydiae* were only present in *Shank3αβ* KO mice, while the mount of *Proteobacteria* and *Verrucomicrobia* was lower. Interestingly, in humans, a significant increase in the *Actinobacterium* phylum was found in patients with ASD [40]. Our results are also in line with the previous reported alterations in gut microbiota of *Shank3* KO mice [16], where similar to our results, an increase in *Actinobacteria* was reported. The consistent increase in *Actinobacteria* in two distinct *Shank3* KO models housed in different animal facilities therefore arises as consistent pattern with relation to ASD in humans.

On genus level, we found a significant increase in *Enterorhabdus* and *Mucispirillum* in *Shank3αβ* KO mice. Both genera contain species that have been associated with both inflammatory markers and active colitis [36,41]. In particular, *Mucispirillum* expansion has been observed during intestinal inflammation [41]. In addition, we observed an increase in the *Clostridium* genus. Bacteria of this genus are major producers of toxins and an increase has been reported in ASD [40,42]. Further, *Shank3αβ* KO mice show significantly higher levels of *Parasutterella. Parasutterella* were reported to be characteristic for patients with Crohn’s disease [43] but they also emerged as significantly associated with children with Autism and functional gastrointestinal disorders that experience abdominal pain [44]. Among the genera significantly decreased in *Shank3αβ* KO mice were *Akkermansia. Akkermansia* (e.g., *Akkermansi muciniphila*) were found low in feces of children with autism [45]. In general, a picture emerges where the observed alterations in the microbiota composition of *Shank3αβ* KO mice support a model of increased inflammation.

In ASD, dysbiosis of microbiota has been associated with a disruption of the mucosal barrier leading to alteration in the intestinal permeability [46], which may cause a change in the inflammatory status of mice, a major process in the interaction between gut and brain. In our study, we could confirm the previously reported expression of *Shank3* in gut epithelial cells [12]. Loss of *Shank3αβ* in mice not only produces a phenotype in nervous tissue. Here, we report abnormal GI morphology. The length of villi was reduced in *Shank3αβ* KO mice and ZONULIN expressed at significantly higher level.

Increased intestinal permeability has been recently proposed to play a key role in the pathogenesis of chronic inflammatory diseases [32], such as irritable bowel syndrome [47], but also ASD [48]. Since the intestinal epithelium provides the interface between host and environment, inappropriate antigen trafficking through the intestinal mucosa may occur upon increased intestinal permeability. While under normal physiological conditions, the majority of antigens passes through the transcellular pathway, where lysosomal degradation produces small non-immunogenic peptides, only ∼10% of proteins cross the epithelium through the paracellular pathway as full intact proteins or partially digested peptides. This results in antigenic tolerance, which can be severely affected in case this ratio changes [32]. Intestinal permeability in turn is tightly connected to microbiota composition. ZONULIN is a major regulator of intestinal permeability and an increase in ZONULIN as observed here in *Shank3αβ* KO mice has been associated with increased permeability [49].

In line with this, we could show that bacterial LPS accumulates in significantly higher amount in the liver of *Shank3αβ* KO mice. Together, these results hint towards a disruption of the mucosal barrier.

We could further confirm the increased expression of inflammatory markers in *Shank3* KO mice. In particular, we observed significantly higher IL-6 levels in the capillary network within the brain of *Shank3αβ* KO mice, likely reflecting higher systemic IL-6 levels. Increased serum cytokine levels in autistic patients have previously been modeled in mice by maternal immune activation (MIA). MIA results in the production of inflammatory cytokines leading to neurological and immunological disturbances in the offspring resulting in autism-like behavioral deficits. These studies have pointed towards IL-6 as a key cytokine involved in these events [25]. During inflammation, IL-6 was shown to induce the expression of other cytokines and immune regulatory genes. In addition, IL-6 can initiate the transcription of neural regulatory genes [25]. Intriguingly, administration of an anti-IL-6 antibody in MIA mice rescued some of the behavioral deficits.

The increased levels of IL-6 were accompanied by significantly increased number of GFAP positive cells in the brain of *Shank3αβ* KO mice. GFAP expression is a marker for astrogliosis [50] and increased levels of GFAP expression in cortex were also reported in some human individuals with ASD [51].

Taken together, in line with our previous report on SHANK3 in the GI tract [12] the presence of other “synaptic” proteins in the GI epithelium makes the existence of a protein complex similar to the one described at excitatory postsynapses in enterocytes more than likely. The loss of this complex may lead to morphological and functional abnormalities in the GI tract, ultimately resulting in alterations in the microbiome and the passage of bacterial metabolites and compounds into the host animal. While our data shows correlation and not causation, these molecules may act as trigger for immune responses leading to increased levels of cytokines, among them IL-6 causing an inflammatory response that ultimately will affect brain development and function, e.g., via the activation of astrocytes. Thus, the contribution of extracerebral factors to the phenotype of SHANK3 deficient mice and humans is likely. The possibility of specific interventions to alter the microbiome may provide new vistas for novel therapeutic approaches such as dietary manipulations in ASD.

## 4. Materials and Methods

### 4.1. Materials

Paraformaldehyde was purchased from Merck and D-Saccharose was from Roth, Karlsruhe, Germany. Alexa Fluor conjugated secondary antibodies and ProLong^®^ Gold antifade reagent from Invitrogen/Life Technologies Europe, Darmstadt, Germany. Zonulin 1 antibody was purchased from Thermo Fisher Scientific (Invitrogen) (Waltham, MA, USA); Claudin3 antibody from Abcam (Berlin, Germany); FABP2 antibody from Thermo Fisher Scientific (Invitrogen); LPS antibody from Origene (Rockville, MD, USA); and IL6 antibody was purchased from Cell signaling Technologies (Danvers, MA, USA); GFAP antibody was purchased from Sigma Aldrich (St. Louis, MO, USA); Cytokeratin and Vimentin antibody from Abcam. For SHANK3 western blotting in-house polyclonal rabbit SHANK3 antibodies were used that have been described previously [27,52]. iScriptTM cDNA Synthesis Kit, SSoAdvanced Universal SYBR^®^ Green Supermix and customized PrimePCR plates were purchased from Bio-Rad, Hercules, CA, USA. QuantiTect Primer Assays, RNeasy Mini Kit and QuantiFastTM SYBR_Green RT-PCR kit were purchased from Qiagen, Hilden, Germany. Unless otherwise indicated, all other chemicals were obtained from Sigma-Aldrich.

### 4.2. Animals

*Shank3αβ* mutants were published and characterized before and raised on a C57BL/6 background [27]. All animal experiments were performed in compliance with the guidelines for the welfare of experimental animals issued by the Federal Government of Germany and approved by the Regierungspraesidium Tuebingen and the local ethics committee (Ulm University) (project code - O.103, date of approval May 12th 2016). Both wild type and *Shank3αβ* KO mice received the same standard laboratory diet (ssniff GmbH, Germany) and consumed similar amounts of food and water that was accessed ad libitum.

### 4.3. Microbiome Analysis

DNA extraction—DNA extraction of murine fecal samples was performed using the Mo Bio PowerFecal DNA Isolation Kit (Qiagen, Hilden, Germany) according to the manufacturer´s protocol. After elution, the resulting DNA concentration was measured on the Nanodrop 2000 (Thermo Fisher Scientific, Waltham, MA, USA). Purity was assessed by calculating the measured A260/A280 ratio using a Nanodrop. DNA samples with an A260/A280 ratio in between 1.7 to 2.0 were considered pure and used for MiSeq.

Pyrosequencing of 16S rDNA region V3 to V5—Primers were designed to target conserved sequences around the variable region 3 to 5 (V3 to V5) of bacterial 16S rDNA. 16s Microbiome Profiling with MiSeq was performed by Eurofins Genomics (Ebersberg, Germany).

Pyrosequencing data processing and taxonomic classification—Data processing and taxonomic classification was performed by Eurofins Genomics. In brief, after removing all reads with errors, the remaining reads were processed using minimum entropy decomposition (MED), thereby partitioning the marker gene dataset into OTUs (Operational Taxonomic Units). Taxonomic information was assigned to each OTU by BLAST alignments of representative cluster sequences to the NCBI database. A specific taxonomic assignment for each OUT was transferred from a set of best-matching reference sequences. Only reference sequences with an 80% sequence identity across at least 80% of the sequence were considered for reference purposes. Sequences were not assigned to an OTU if they were considered as noise according to the OTU picking algorithm (including potential chimeric sequences and singletons). OTU and taxonomic assignments were further processed with the QIIME software package (version 1.8.0, http://qiime.org/). Normalization after Angly [53] of bacterial and archaea taxonomic assignment abundance with lineage specific copy numbers of marker genes was performed for estimate improvement. Therefore, the number of reads assigned to one species was divided by a known or assumed number of marker regions/genes.

Statistical analysis was performed testing for significance without correction for multiple comparisons due to the low number of simultaneous tested hypotheses. Correction for multiple comparisons does not alter results reported in the manuscript with the exception of differences observed for Proteobacteria on phylum level.

### 4.4. Immunohistochemistry

Paraffin-embedded sections of small intestine were cut at 4.5 to 5 μm thickness. Afterwards, sections were treated with Xylene 2× for 5 min each and submerged in 100%, 90%, 70% Ethanol and H_2_O for 5 min each. Sections were treated in 10 mM sodium citrat buffer pH 6.0 for 15 min around boiling point (Microwave at 600 W). The slides were cooled down to room temperature (RT) for approximately 30 min und washed two times in PBS for 2 min each. The tissue on each slide was surrounded with a fat tissue stick. To avoid unspecific antibody binding the tissue was blocked with blocking solution (BS) (10% FBS in 1× PBS) for 1 h at RT. Subsequently, the tissue was incubated with primary antibody diluted in BS for 2 h at RT in a humid chamber. After washing 3× with PBS for 5 min, the tissue was incubated with secondary antibody, diluted in BS, for 1 h in a humid chamber, followed by a wash step with PBS for 5 min. The tissue was counterstained with DAPI (4′,6-Diamidin-2-phenylindol).

Frozen brain sections were cut at 14 μm thickness. After cryosections were thawed for 20 min in a hydrated staining chamber, sections were fixed in 4% paraformaldehyde (PFA)/4% sucrose/PBS for 20 min and washed three times in PBS for 5 min each. Subsequently, sections were treated with 1× PBS with 0.2% Triton X-100 for 20 min at RT and 1× PBS with 0.05% Triton X-100 for 10 min at RT. To avoid unspecific antibody binding blocking was performed with blocking solution (BS) (10% FBS in 1× PBS) for 1 h at RT. Afterwards, the tissue was incubated with primary antibody diluted in BS overnight at 4 °C in a humid chamber. The following day after washing with 1× PBS with 0.05% Triton X-100 for 10 min, the tissue was incubated with secondary antibody coupled to alexa488 or alexa568, diluted in BS, for 2 h at 37 °C in a dark humid chamber, followed by a 3× wash steps with 1× PBS with 0.05% Triton X-100 for 5 min each and a 5 min wash step with 1× PBS. The tissue was counterstained with DAPI (4′,6-Diamidin-2-phenylindol) for 5 min at RT, washed with aqua bidest before being mounted with Vecta Mount. Fluorescence images were obtained using an inverted confocal microscope (Zeiss LSM710, Göttingen, Germany) and an ImageXpress Micro Spinning Disc Confocal High-Content Imaging System (Molecular Devices, San Jose, CA, USA), and analyses of signal intensities were performed with ImageJ 1.48r.

### 4.5. Histology

Paraffin-embedded sections from intestine were obtained of small intestine from adult mice (10 weeks of age). From each intestine, 4 cm were fixed in 4% buffered formalin. Per sample, three small parts were embedded in paraffin wax longitudinally (for cross sections) and horizontally (for longitudinal sections). For morphological analyses, small intestinal sections were cut at 2 µm and stained with Haematoxylin/Eosin (HE) or the periodic acid–Schiff (PAS)-reaction. Immunohistochemistry staining of 4.5 to 5 μm sections was performed using the Benchmark XT Autostainer (Ventana Medical systems, Tucson, USA). All required reagents were purchased from Ventana. Dilution of primary antibodies was done according to the respective manufacturer’s recommendations. For detection of primary antibody the OptiView DAB IHC Detection Kit or the ultra universal Alkaline Phosphatase Red Kit was used. Additionally, sections were washed in water, lightly counterstained with Haematoxylin, dehydrated, and mounted. Images were obtained using the Mirax Desk scanner and the MIRAX Viewer 1.12.22.0 software (Zeiss, Göttingen, Germany).

### 4.6. qRT-PCR

Total RNA was isolated with the RNeasy Mini Kit according to the manufacturer’s protocol. All of the optional purification steps were performed and RNA eluted with sterile RNAse-free water.

cDNA synthesis of pooled RNAs was performed with the iScript^TM^ cDNA Synthesis Kit (Bio-Rad) according to the manufacturer’s protocol in a total reaction volume of 20 µL and a maximum of 1 µg RNA/reaction. Quantitative real-time-PCR was performed using the SSoAdvanced Universal SYBR^®^ Green Supermix (Bio-Rad) and customized PrimePCR plates in 96 well format with immobilized primers (Bio-Rad) according to the manufacturer’s protocol with a final reaction volume of 20 µL and 2 ng cDNA/well. Resulting data were analyzed using the hydroxymethylbilane synthase (*HMBS*) or Glyceraldehyde 3-phosphate dehydrogenase (*GAPDH*) gene as an internal standard to normalize transcript levels. Cycle threshold (*ct*) values were calculated by the CFX Manager (Bio-Rad, version 3.1, Hercules, CA, USA).

Alternatively, first strand synthesis and quantitative real-time-PCR amplification were performed in a one-step, single-tube format using the QuantiFast^TM^ SYBR_Green RT-PCR kit from Qiagen according to the manufacturer’s protocol in a total volume of 20 µL. Thermal cycling and fluorescent detection were performed using the Rotor-Gene Q real-time PCR machine (model 2-Plex HRM) (Qiagen, Hilden, Germany). The SYBR Green I reporter dye signal was measured. Resulting data were analyzed using the *HMBS* gene as an internal standard to normalize transcript levels. Cycle threshold (*ct*) values were calculated by the Rotor-Gene Q Software (Qiagen, version 2.0.2, Hilden, Germany). All quantitative real-time PCR reactions were run in technical triplicates and mean *ct*-values for each reaction were taken into account for calculations.

### 4.7. Protein Biochemistry

To obtain homogenate from GI tissue, small intestinal epithelium was isolated from mesenchyme following a protocol after Nik and Carlsson [28]. In brief, small intestine was cut into 4–5 cm long pieces. Gut mucus was removed by gently squeezing it out of the intestine with the blunt point of tweezers. Each piece was inverted by inserting a rod, securing the intestine at one end with a suture and pulling the rod back. The rod with the inverted piece was inserted into a pipet tip (1000 μL) and one end of the intestine pulled onto the tip. After careful removal of the rod, the second end of the intestinal piece is pinched off with a suture. The inverted intestine with the attached pipet tip was submerged in cell recovery solution and repeatedly inflated and reflated with air over the course of at least 30 min per piece. During this time the epithelium is separated from the other intestinal layers. For lysis, RIPA buffer + PI (Complete EDTA-free Protease Inhibitor Cocktail tablets; Roche, Mannheim, Germany) is applied to the collected mouse tissue. To disrupt the epithelium a sonicator was used (4 pulses, lasting 1 s each). Afterwards the lysate was incubated for 2 h at 4 °C on a rotator followed by centrifugation for 20 min at 4 °C at 11,700 rpm. Protein concentration was determined by Bradford protein assay.

To obtain homogenate from liver tissue, tissue was immersed in Hepes Sucrose buffer (10 mM Hepes, 0.32 M Sucrose) and disrupted using a sonicator (fisherbrand sonic dismembranator 120, Fisher scientific, Hampton, NH, USA). Proteins were separated by SDS-PAGE and blotted onto nitrocellulose membranes (GE Healthcare). Immunoreactivity was visualized using horseradish peroxidase (HRP)-conjugated secondary antibodies and the SuperSignal detection system (Pierce, Thermo Fisher, Waltham, MA, USA).

### 4.8. Statistic

Statistical analysis was performed using Graph Pad Prism 5 (La Jolla, CA, USA), and tested for significance using *t* tests. All values were normally distributed. In experiments using pooled samples or three replicates, normal distribution was not tested but assumed as the most likely scenario. Statistical tests were two tailed with a significance level of α ≤ 0.05. Significances are stated with *p* values <0.05 *; <0.01 **; <0.001 ***.

qRT PCR quantification—Relative quantification is based on internal reference genes to determine virtual mRNA levels of target genes. Cycle threshold (ct) values were calculated by the Rotor-Gene Q Software (version 2.0.2, Qiagen, Hilden, Germany). Ct values were transformed into virtual mRNA levels according to the formula: Virtual mRNA level = 10 * ((ct_(target)_ − ct_(standart)_)/slope of standard curve).

Western blot quantification—Evaluation of bands from Western blots (WBs) was performed using ImageJ. Three independent experiments were performed and blots imaged using a UVITEC Alliance Q9 Advanced system (Cleaver scientific, Rugby, UK). The individual bands were selected and the integrated density was measured. All WB bands were normalized to β-Actin and the ratios averaged and tested for significance.

## Supplementary Materials

Supplementary materials can be found at https://www.mdpi.com/1422-0067/20/9/2134/s1.
