# Supplementary material for: Altered Intestinal Morphology and Microbiota Composition in the Autism Spectrum Disorders Associated SHANK3 Mouse Model
Source: Int J Mol Sci. 2019 Apr 30;20(9):2134. doi: 10.3390/ijms20092134 (PMC6540607; doi:10.3390/ijms20092134)
Supplement: Supplementary file 1 [file ijms-20-02134-s001.pdf]

International Journal of *Molecular Sciences*

MDPI

Article

Altered intestinal morphology and microbiota composition in the Autism Spectrum Disorders associated SHANK3 mouse model

Ann Katrin Sauer, Juergen Bockmann, Konrad Steinestel, Tobias M. Boeckers and Andreas M. Grabrucker

Figure Supplements: Figure S1

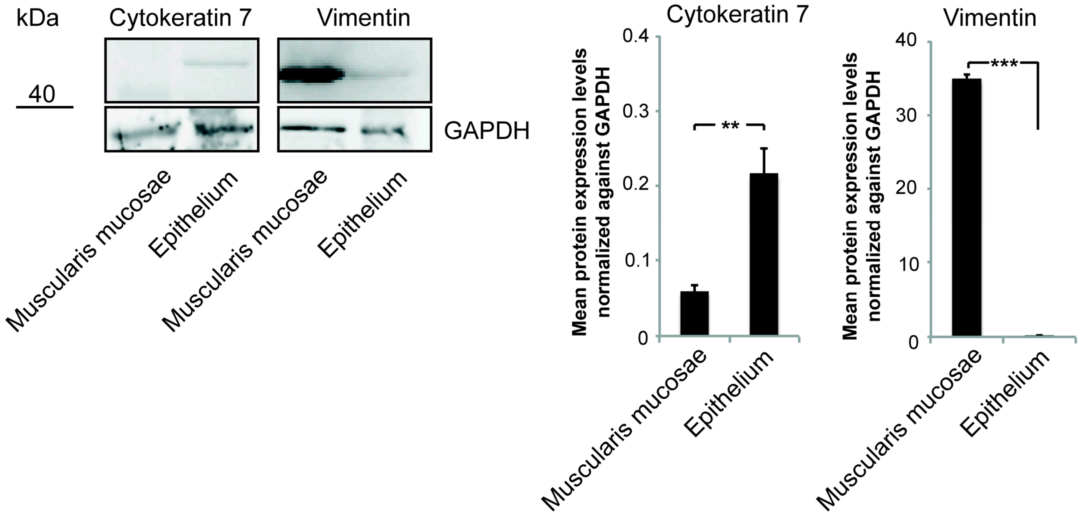

Figure supplement 1. Purification and analysis of GI epithelium of wildtype and *Shank3αβ* KO mice. Western Blot analysis for the expression of Vimentin, and Cytokeratin 7 shows that epithelium can be successfully separated from gut muscle (t-test, *Cytokeratin7*: $p < 0.01$; *Vimentin*: $p < 0.001$ (n = 3 western blot bands were analyzed from 3 animals)). ** $p < 0.01$; *** $p < 0.001$

Figure Supplements: Figure S2

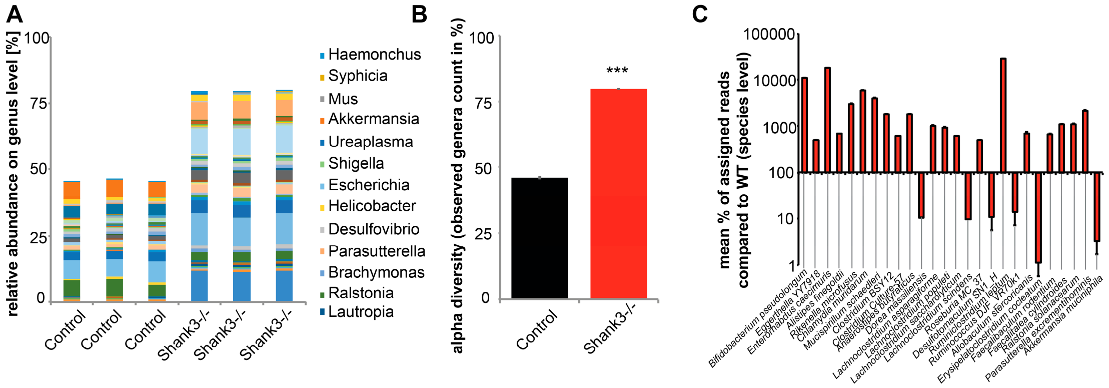

Figure supplement 2. (A) Bar plot graph comparing Control and *Shank3αβ* KO mice on genus level. Within group sample differences were small compared to between group differences. **(B)** Measurement of alpha diversity reveals a significant difference between Controls and *Shank3αβ* KO mice (*t*-test, $p < 0.0001$). **(C)** Identification of species by 16S rRNA sequencing can only be done with low confidence. Species with more than 5 fold increase or decrease in *Shank3αβ* KO mice compared to Control are shown. Among those highly upregulated are: *Bifidobacterium pseudolongum, Eggerthella YY7918, Enterorhabdus caecimuris, Alistipes finegoldii, Rikenella microfusus, Chlamydia muridarum, Mucispirillum schaedleri, Clostridium BSY12, Clostridium Culture-57, Anaerostipes butyraticus, Lachnoclostridium asparagiforme, Lachnoclostridium populeti, Lachnoclostridium saccharolyticum, Roseburia MC_37, Ruminiclostridium leptum, Allobaculum stercoricanis, Faecalibaculum rodentium, Faecalitalea cylindroides, Ralstonia solanacearum, Parasutterella excrementihominis.* Species significantly downregulated include: *Dorea massiliensis, Lachnoclostridium scindens, Desulfotomaculum SN1_H, Ruminococcus DJF_VR70k1, Erysipelatoclostridium cocleatum, Akkermansia muciniphila.* *** $p < 0.001$
